# CITEdb: a manually curated database of cell–cell interactions in human

**DOI:** 10.1093/bioinformatics/btac654

**Published:** 2022-09-30

**Authors:** Nayang Shan, Yao Lu, Hao Guo, Dongyu Li, Jitong Jiang, Linlin Yan, Jiudong Gao, Yong Ren, Xingming Zhao, Lin Hou

**Affiliations:** School of Statistics, Capital University of Economics and Business, Beijing 100070, China; Department of Industrial Engineering, Center for Statistical Science, Tsinghua University, Beijing 100084, China; MOE Key Laboratory of Bioinformatics, School of Life Sciences, Tsinghua University, Beijing 100084, China; State Key Laboratory of Translational Medicine and Innovative Drug Development, Jiangsu Simcere Diagnostics Co., Ltd, Nanjing 210042, China; Nanjing Simcere Medical Laboratory Science Co., Ltd, Nanjing 210042, China; Department of Industrial Engineering, Center for Statistical Science, Tsinghua University, Beijing 100084, China; MOE Key Laboratory of Bioinformatics, School of Life Sciences, Tsinghua University, Beijing 100084, China; Department of Mathematics, University of Michigan, Ann Arbor, MI 48109, USA; State Key Laboratory of Translational Medicine and Innovative Drug Development, Jiangsu Simcere Diagnostics Co., Ltd, Nanjing 210042, China; Nanjing Simcere Medical Laboratory Science Co., Ltd, Nanjing 210042, China; State Key Laboratory of Translational Medicine and Innovative Drug Development, Jiangsu Simcere Diagnostics Co., Ltd, Nanjing 210042, China; Nanjing Simcere Medical Laboratory Science Co., Ltd, Nanjing 210042, China; State Key Laboratory of Translational Medicine and Innovative Drug Development, Jiangsu Simcere Diagnostics Co., Ltd, Nanjing 210042, China; Nanjing Simcere Medical Laboratory Science Co., Ltd, Nanjing 210042, China; Key Laboratory of Computational Neuroscience and Brain-Inspired Intelligence (LCNBI) and ZJLab, Wuxi, China; Department of Industrial Engineering, Center for Statistical Science, Tsinghua University, Beijing 100084, China; MOE Key Laboratory of Bioinformatics, School of Life Sciences, Tsinghua University, Beijing 100084, China

## Abstract

**Motivation:**

The interactions among various types of cells play critical roles in cell functions and the maintenance of the entire organism. While cell–cell interactions are traditionally revealed from experimental studies, recent developments in single-cell technologies combined with data mining methods have enabled computational prediction of cell–cell interactions, which have broadened our understanding of how cells work together, and have important implications in therapeutic interventions targeting cell–cell interactions for cancers and other diseases. Despite the importance, to our knowledge, there is no database for systematic documentation of high-quality cell–cell interactions at the cell type level, which hinders the development of computational approaches to identify cell–cell interactions.

**Results:**

We develop a publicly accessible database, CITEdb (Cell–cell InTEraction database, https://citedb.cn/), which not only facilitates interactive exploration of cell–cell interactions in specific physiological contexts (e.g. a disease or an organ) but also provides a benchmark dataset to interpret and evaluate computationally derived cell–cell interactions from different tools. CITEdb contains 728 pairs of cell–cell interactions in human that are manually curated. Each interaction is equipped with structured annotations including the physiological context, the ligand–receptor pairs that mediate the interaction, etc. Our database provides a web interface to search, visualize and download cell–cell interactions. Users can search for cell–cell interactions by selecting the physiological context of interest or specific cell types involved. CITEdb is the first attempt to catalogue cell–cell interactions at the cell type level, which is beneficial to both experimental, computational and clinical studies of cell–cell interactions.

**Availability and implementation:**

CITEdb is freely available at https://citedb.cn/ and the R package implementing benchmark is available at https://github.com/shanny01/benchmark.

**Supplementary information:**

Supplementary data are available at *Bioinformatics* online.

## 1 Introduction

Cells are basic units of multicellular organisms, which cooperate with each other to carry out complex physiological functions. Some cells interact directly with others, mainly mediated by gap junctions, cell adhesions and ligand–receptor interactions of membrane proteins (Bennett *et al.*, 1991; Bosenberg and Massagué, 1993; Singer, 1992). Others form indirect cell–cell interactions, including autocrine, paracrine and endocrine secretion, are mediated by soluble factors (Byrne *et al.*, 2014) and extracellular vehicles (Camussi *et al.*, 2010). Researches on cell–cell interactions have broadened our understanding of how cells work together, which further enhances applications including cell culturing (Xu *et al.*, 2010), tissue regeneration (Grellier *et al.*, 2009), and most importantly, therapies targeting cell–cell interactions for cancers and other diseases (Davies and Holgate, 2002; Dominiak *et al.*, 2020; Song *et al.*, 2019).

Despite the importance of cell–cell interactions, their documentation has been limited, and relevant resources are listed below. Cytokine networks between immune and body cells (Frankenstein *et al.*, 2006) were established by extracting cytokine connections from two Internet cytokine databases. Cell Interaction Knowledgebase (Chen *et al.*, 2007) focuses on cell–cell interactions between immune-related cells, including macrophages and dendritic cells. EndoNet (Dönitz *et al.*, 2008) provides intercellular communications mediated by hormones and hormone receptors. Moreover, a relevant resource is databases for experimentally identified and computationally predicted ligand–receptor pairs (Ramilowski *et al.*, 2015; Shao *et al.*, 2020), which have been frequently used to predict cell–cell interactions in single-cell RNA-seq (scRNA-seq) analysis (Almet *et al.*, 2021; Armingol *et al.*, 2021; Shao *et al.*, 2020). To our knowledge, the databases of cell–cell interactions at the cell type level are very limited.

To address these limitations, we developed a database (CITEdb) that documents literature curated high-quality cell–cell interactions at cell type level. The interactions are manually curated from the literature and annotated with comprehensive information. We searched related publications in PubMed and manually extracted the sentences describing cell–cell interactions. The contexts of cell–cell interactions and other available information (e.g. the function of the interaction and the method used to obtain the interaction) were recorded. The above information was further processed to be structured and unified. A web interface of CITEdb (https://citedb.cn/) is freely available, which provides the full database as an interactive table, as well as a ‘Search’ page for a customized search of cell–cell interactions. CITEdb can serve as a convenient tool to explore and validate cell–cell interactions, which may be especially useful for single-cell studies.

## 2 Data collection

Cell–cell interactions in human were extracted from the literature. We obtained 574 publications (Supplementary Table S1) from the PubMed by searching a list of keywords regarding the title and abstract, such as ‘cell to cell interaction(s)’, ‘cell–cell interaction(s)’, ‘intercellular interaction(s)’, ‘cell to cell communication(s)’, ‘cell–cell communication(s)’ or ‘intercellular communication(s)’ together with ‘human(s)’ and ‘cell type(s)’. Full texts are accessible for 509 of the publications. We carefully read the main texts, tables and figures to extract the cell types involved in cell–cell interactions. In addition, we collected details of the interactions, whenever they are available in the curated publications, including the contexts of the cell–cell interactions, when (e.g. acute phase) and where (i.e. tissue) the interactions take effect, the interaction mediators, the experimental approaches and the corresponding biological functions. PubTator (Wei *et al.*, 2019) was used to highlight the biomedical entities including species, diseases and genes, which could assist efficient information extraction. Explicit criteria to nail a cell–cell interaction and to determine its direction are provided in Supplementary Note S1.1.

## 3 Processing and annotation

The names of cell types have been unified by adopting the categorization of CellMarker, including cell type level and class level (Zhang *et al.*, 2019). For example, T helper1 cell, T helper2 cell, CD4+ T cell, CD8+ T cell and other congeneric cells are cell types, while they are classified as T cell at class level in CITEdb. For interaction contexts, usually a disease or an organ, we map the descriptions to at least one MeSH term via ‘MeSH on Demand’. Subsequently, MeSH terms were grouped into several categories based on the MeSH tree structures. For example, the disease ‘asthma’ was grouped into respiratory tract diseases and immune system diseases. For the Method column, the method details of obtaining cell–cell interactions were refined into ‘computational’ and ‘experimental’. In cases that an interaction is discussed without any reference or experimental evidence, it is labelled as ‘not sure’.

## 4 Data statistics

Currently, CITEdb contains 728 cell–cell interactions in human, involving the 204 physiological contexts, with a notable ascending trend in the number of related publications (Supplementary Fig. S1A). Cell–cell interactions were the most frequently discussed in contexts such as ‘immune response’, ‘bone microenvironment’, ‘carcinogenesis’ and ‘breast cancer’ (Supplementary Fig. S1).

## 5 Web interface

**Fig. 1.**
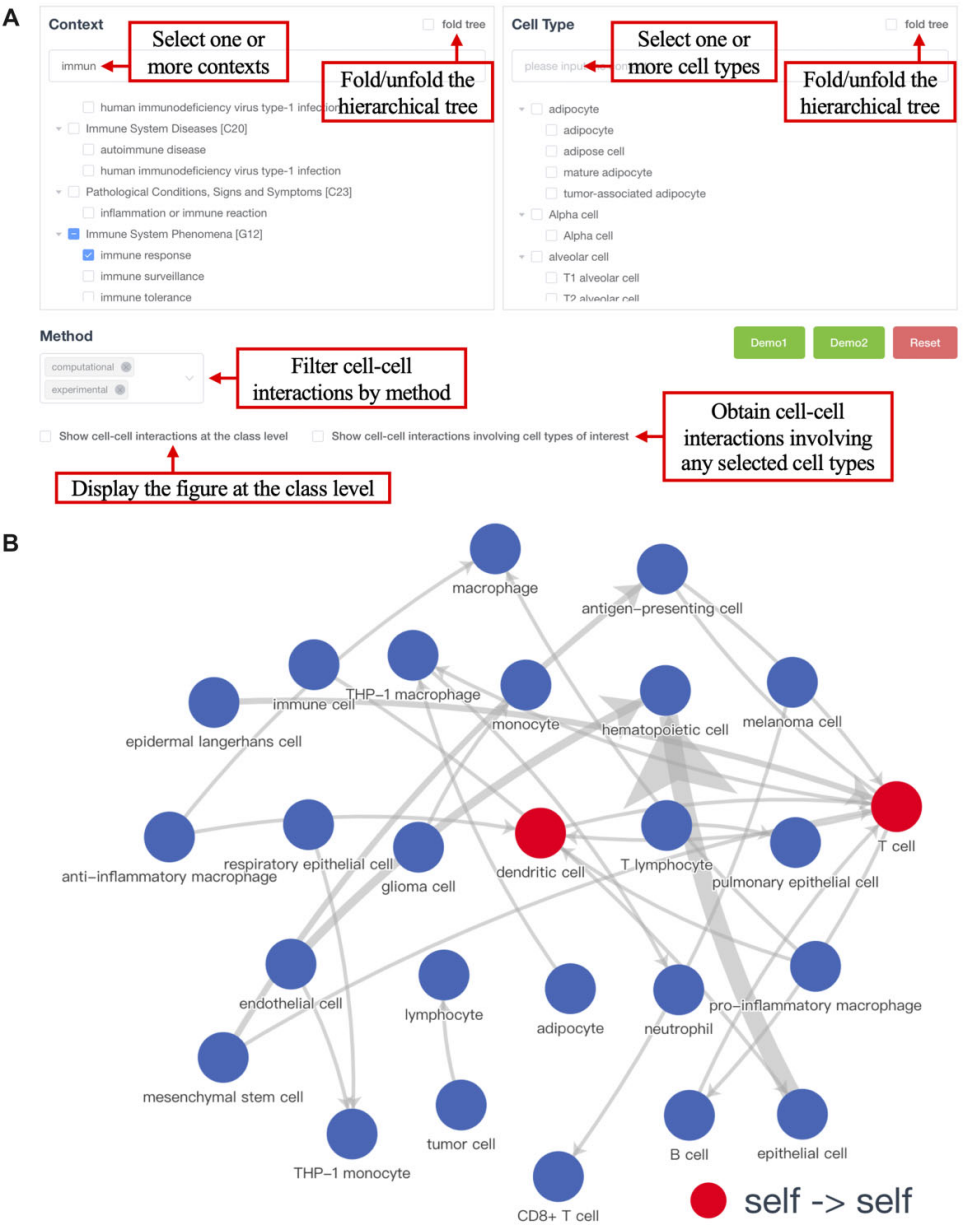
Overview of the CITEdb website. (**A**) Users can search for cell–cell interactions by selecting the contexts or the cell types of interest, as well as selecting contexts and cell types simultaneously. (**B**) Graphical display of the search result. Nodes represent cell types, and edges represent cell–cell interactions. The thickness of edges indicates the number of evidences supporting the interaction

CITEdb provides an interactive web interface to search, visualize and download. In the ‘Search’ page, users can search for cell–cell interactions in contexts or cell types of interest by selecting one or more terms from the corresponding hierarchical tree. Alternatively, users can search by keyword (case sensitive), which is a substring of a context or a cell type. A Demo of the search function of CITEdb can be viewed by clicking on the ‘Demo1’ button (Fig. 1A). The search returns a graph with cell types as nodes and cell–cell interactions as edges, with the thickness of edges indicating the number of evidences supporting the interaction (Fig. 1B). Note that the thickness is not a measure of the reliability of the interaction. We have implemented interactive features on the website. When browsing the search result, users can obtain information of mediators involved in the cell–cell interactions by clicking on the edges. Plus, the number of interactions annotated with the cell types is revealed by clicking on the nodes. In addition to the graph, an interactive table of the searched cell–cell interactions is also provided. By clicking on the hyperlink in the MeSH term, it will navigate to details of the term. Users can further filter the interactions by selecting methods from the pull-down menu (Fig. 1A). While cell–cell interactions between the selected cell types is returned by default, users can obtain cell–cell interactions involving any selected cell types by checking the box of ‘Show cell–cell interactions involving cell types of interest’ (Fig. 1A). Additionally, users can opt to show the interactions at class level by checking the box of the ‘Show cell–cell interactions at the class level’ (Fig. 1A), for which a line is drawn when any cell type in one class has an interaction with any cell type in the other class. A similar demonstration of searching by cell types is provided in ‘Demo2’ (Fig. 1A). The figures and tables are downloadable. Moreover, the entire dataset can be obtained from the ‘Download’ page. More descriptions, such as the specific meaning of the column names, and guidance on using the database can be found on the ‘Help’ page.

## 6 Applications

We describe two application scenarios to showcase how CITEdb facilitates research in cell–cell interactions. In Scenario I, a scientist wants to identify cell–cell interactions related to immune response. By selecting the context ‘immune response’ in the ‘Search’ page, they can obtain 37 interactions involving in 26 cell types, with a graphical display (Fig. 1B) and a table with detailed information. Among those, 29 interactions are annotated with mediators, in which 10 are mediated by ligand–receptors interactions. For example, the search revealed that the interaction between epithelial cells and haematopoietic cells is mediated by multiple ligand–receptor pairs (Ramilowski *et al.*, 2015). A similar example queried by cell types is provided in Supplementary Note S1.2.

**Fig. 2.**
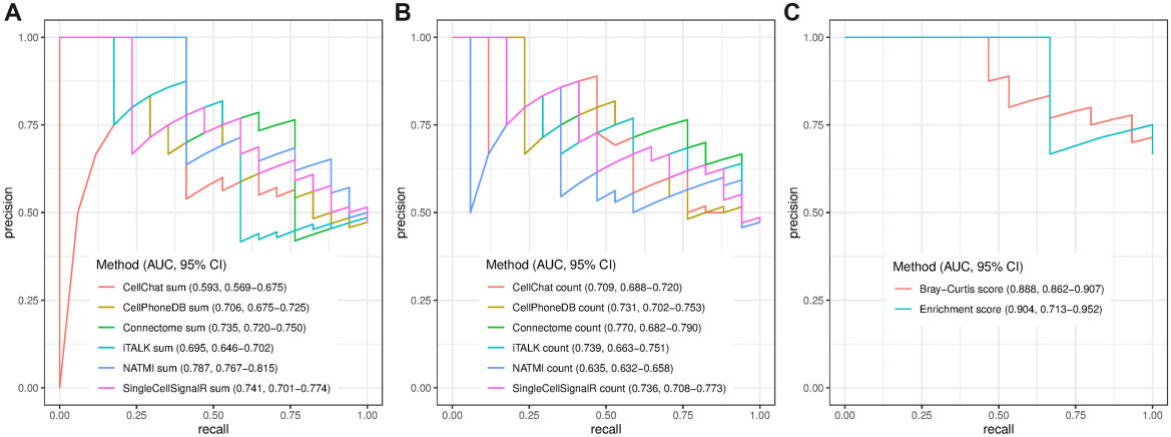
Precision-recall curves in evaluating various algorithms that predict cell–cell interactions from a human metastatic melanoma scRNA-seq dataset. CITEdb interactions are used as a benchmark dataset. The directional interactions are predicted by six algorithms and then summarized into cell level interactions by the sum of communication scores (**A**) and the count of active LR pairs (**B**). The unidirectional interactions are inferred by Bray–Curtis score and the enrichment score (**C**). The area under the curve (AUC) is presented with 95% confidence interval (CI)

In Scenario II, a biologist wants to identify cell–cell communications from their scRNA-seq dataset, and they predicted cell–cell interactions with various computational tools. At this point, they wonder: Have the predicted interactions been reported in literature? Which of the identified interactions represent novel findings? CITEdb can provide an initial benchmark dataset to interpret and evaluate the computational inferred cell–cell interactions. For illustration, we downloaded a scRNA-seq dataset of melanoma (Tirosh *et al.*, 2016), containing 4645 cells and 23 686 genes from 19 melanoma samples. The non-malignant cell types include B cell, endothelial cell, cancer-associated fibroblast, macrophage, natural killer cell and T cell. Of the 36 candidate cell–cell interactions between these cell types (6 self-interactions plus 30 directed interactions), all 6 self-interactions and 15 of the directed interactions (corresponding to 9 undirected interactions) were documented at the class level in CITEdb. We used six methods to infer cell–cell interaction networks for the dataset, namely SingleCellSignalR (Cabello-Aguilar *et al.*, 2020), CellPhoneDB (Efremova *et al.*, 2020), CellChat (Jin *et al.*, 2021), Connectome (Raredon *et al.*, 2022), iTALK (https://github.com/Coolgenome/iTALK) and NATMI (Hou *et al.*, 2020). They were implemented by directly running LIANA (Dimitrov *et al.*, 2022) with its built-in consensus resource for ligand–receptor pairs. The results of the algorithms were then summarized into cell–cell interactions by two approaches, the sum of communication scores (sum) and the count of active ligand–receptor pairs (count). Two algorithms that predict unidirectional interactions are also considered, the Bray–Curtis score (https://github.com/earmingol/cell2cell) and the enrichment score (Krausgruber *et al.*, 2020). We calculated precision-recall (PR) curves and the area under the PR curve at the cell type class level. The details of implementing the algorithms and computing the PR curves with 95% confidence intervals were described in Supplementary Note S1.3. We have also provided an R package for the benchmark analysis (https://github.com/shanny01/benchmark). For the directed predictions, the algorithms that achieved the best performance are NATMI and SingleCellSignalR when combined with the sum approach, and Connectome when combined with the count approach (Fig. 2A and B). For the unidirectional predictions, Bray–Curtis score and the enrichment score achieved comparable performance (Fig. 2C). By exploring CITEdb, users can quickly gather the existing evidence to interpret their results.

## 7 Discussion

CITEdb serves as a resource facilitating the search for cell–cell interactions with a user-friendly interface for customized exploration, which is the first attempt to catalogue cell–cell interactions at cell type level. The work is beneficial to both experimental, computational and clinical studies involving cell–cell interactions. There are several limitations in this work. First, although we put a lot of effort to comprehensively curate literature, some cell–cell interactions might be missed due to restricted access to full text or incomplete coverage of search keyword. Second, due to the bias in research resources, our database inevitably contains more interactions in frequently studied physiological contexts. Third, although ligand–receptor pair information is essential in cell communications, in CITEdb we focus on the cell type level interactions. We refer interested users to existing resources of ligand–receptor pairs (Cabello-Aguilar *et al.*, 2020; Efremova *et al.*, 2020; Shao *et al.*, 2020; Jin *et al.*, 2021). Finally, we would like to point out future directions to improve the documentation of cell–cell interactions. Interactions of other species, such as mouse, rat and drosophila, will be of interest. Text mining-based methods need to be developed to extract cell–cell interactions from literature.

## Supplementary Material

btac654_Supplementary_Data

## Data Availability

The data underlying this article can be obtained at https://citedb.cn/ and https://github.com/shanny01/benchmark/tree/main/data.

## References

[btac654-B1] Almet A.A. et al (2021) The landscape of cell–cell communication through single-cell transcriptomics. Curr. Opin. Syst. Biol., 26, 12–23. 33969247 10.1016/j.coisb.2021.03.007 PMC8104132

[btac654-B2] Armingol E. et al (2021) Deciphering cell–cell interactions and communication from gene expression. Nat. Rev. Genet., 22, 71–88. 33168968 10.1038/s41576-020-00292-x PMC7649713

[btac654-B4] Bennett M.V. et al (1991) Gap junctions: new tools, new answers, new questions. Neuron, 6, 305–320. 1848077 10.1016/0896-6273(91)90241-q

[btac654-B5] Bosenberg M.W. , Massagué J. (1993) Juxtacrine cell signaling molecules. Curr. Opin. Cell Biol., 5, 832–838. 7694603 10.1016/0955-0674(93)90032-l

[btac654-B6] Byrne M.B. et al (2014) Microfluidic platform for the study of intercellular communication via soluble factor-cell and cell-cell paracrine signaling. Biomicrofluidics, 8, 044104. 25379089 10.1063/1.4887098 PMC4189157

[btac654-B7] Cabello-Aguilar S. et al (2020) SingleCellSignalR: inference of intercellular networks from single-cell transcriptomics. Nucleic Acids Res., 48, e55. 32196115 10.1093/nar/gkaa183 PMC7261168

[btac654-B8] Camussi G. et al (2010) Exosomes/microvesicles as a mechanism of cell-to-cell communication. Kidney Int., 78, 838–848. 20703216 10.1038/ki.2010.278

[btac654-B9] Chen S. et al (2007) Cell interaction knowledgebase: an online database for innate immune cells, cytokines and chemokines. In Silico Biol., 7, 569–574. 18467769

[btac654-B10] Davies D.E. , Holgate S.T. (2002) Asthma: the importance of epithelial mesenchymal communication in pathogenesis. Inflammation and the airway epithelium in asthma. Int. J. Biochem. Cell Biol., 34, 1520–1526. 12379273 10.1016/s1357-2725(02)00048-1

[btac654-B11] Dimitrov D. et al (2022) Comparison of resources and methods to infer Cell-Cell communication from single-cell RNA data. Nat. Commun., 13, 3224. 35680885 10.1038/s41467-022-30755-0 PMC9184522

[btac654-B12] Dominiak A. et al (2020) Communication in the cancer microenvironment as a target for therapeutic interventions. Cancers, 12, 1232. 10.3390/cancers12051232 PMC7281160 32422889

[btac654-B13] Dönitz J. et al (2008) EndoNet: an information resource about regulatory networks of cell-to-cell communication. Nucleic Acids Res., 36, D689–694. 18045786 10.1093/nar/gkm940 PMC2238947

[btac654-B14] Efremova M. et al (2020) CellPhoneDB: inferring cell-cell communication from combined expression of multi-subunit ligand-receptor complexes. Nat. Protoc., 15, 1484–1506. 32103204 10.1038/s41596-020-0292-x

[btac654-B15] Frankenstein Z. et al (2006) The immune-body cytokine network defines a social architecture of cell interactions. Biol. Direct., 1, 32. 17062134 10.1186/1745-6150-1-32 PMC1636025

[btac654-B16] Grellier M. et al (2009) Cell-to-cell communication between osteogenic and endothelial lineages: implications for tissue engineering. Trends Biotechnol., 27, 562–571. 19683818 10.1016/j.tibtech.2009.07.001

[btac654-B17] Hou R. et al (2020) Predicting cell-to-cell communication networks using NATMI. Nat. Commun., 11, 5011. 33024107 10.1038/s41467-020-18873-z PMC7538930

[btac654-B18] Jin S. et al (2021) Inference and analysis of cell-cell communication using CellChat. Nat. Commun., 12, 1088. 33597522 10.1038/s41467-021-21246-9 PMC7889871

[btac654-B19] Krausgruber T. et al (2020) Structural cells are key regulators of organ-specific immune responses. Nature, 583, 296–302. 32612232 10.1038/s41586-020-2424-4 PMC7610345

[btac654-B20] Ramilowski J.A. et al (2015) A draft network of ligand-receptor-mediated multicellular signalling in human. Nat. Commun., 6, 7866. 26198319 10.1038/ncomms8866 PMC4525178

[btac654-B21] Raredon M.S.B. et al (2022) Computation and visualization of cell-cell signaling topologies in single-cell systems data using. Sci. Rep., 12, 4187. 35264704 10.1038/s41598-022-07959-x PMC8906120

[btac654-B22] Shao X. et al (2020) CellTalkDB: a manually curated database of ligand-receptor interactions in humans and mice. Brief Bioinformatics, 22, bbaa269. 10.1093/bib/bbaa269 33147626

[btac654-B23] Shao X. et al (2020) New avenues for systematically inferring cell-cell communication: through single-cell transcriptomics data. Protein Cell., 11, 866–880. 32435978 10.1007/s13238-020-00727-5 PMC7719148

[btac654-B24] Singer S.J. (1992) Intercellular communication and cell-cell adhesion. Science, 255, 1671–1677. 1313187 10.1126/science.1313187

[btac654-B25] Song D. et al (2019) Cell-cell communication: old mystery and new opportunity. Cell Biol. Toxicol., 35, 89–93. 30815784 10.1007/s10565-019-09470-y

[btac654-B26] Tirosh I. et al (2016) Dissecting the multicellular ecosystem of metastatic melanoma by single-cell RNA-seq. Science, 352, 189–196. 27124452 10.1126/science.aad0501 PMC4944528

[btac654-B28] Wei C.H. et al (2019) PubTator Central: automated concept annotation for biomedical full text articles. Nucleic Acids Res., 47, W587–W593. 31114887 10.1093/nar/gkz389 PMC6602571

[btac654-B29] Xu J. et al (2010) Cell-cell interaction promotes rat marrow stromal cell differentiation into endothelial cell via activation of TACE/TNF-alpha signaling. Cell Transplant., 19, 43–53. 19796498 10.3727/096368909X474339 PMC2850940

[btac654-B30] Zhang X. et al (2019) CellMarker: a manually curated resource of cell markers in human and mouse. Nucleic Acids Res., 47, D721–D728. 30289549 10.1093/nar/gky900 PMC6323899

