# Correction to: CITEdb: a manually curated database of cell–cell interactions in human

**DOI:** 10.1093/bioinformatics/btad669

**Published:** 2023-11-06

This is a correction to: Nayang Shan, Yao Lu, Hao Guo, Dongyu Li, Jitong Jiang, Linlin Yan, Jiudong Gao, Yong Ren, Xingming Zhao, Lin Hou, CITEdb: a manually curated database of cell–cell interactions in human, *Bioinformatics*, Volume 38, Issue 22, 15 November 2022, Pages 5144–5148, https://doi.org/10.1093/bioinformatics/btac654

In the originally published version of this manuscript, author Xingming Zhao’s affiliation was incorrect.

The author’s affiliation has been corrected online to:

“Key Laboratory of Computational Neuroscience and Brain-Inspired Intelligence, Ministry of Education, Fudan University, Shanghai, China.”

