# Supplementary material for: CITEdb: a manually curated database of cell–cell interactions in human
Source: Bioinformatics. 2022 Sep 30;38(22):5144–8. doi: 10.1093/bioinformatics/btac654 (PMC9665858; doi:10.1093/bioinformatics/btac654)
Supplement: btac654_Supplementary_Data [file btac654_supplementary_data.docx]

**Supplementary Data**

Contents

1 Supplementary Notes2

1.1 Criteria to define a cell-cell interaction2

1.2 An example for querying by cell types3

1.3 Details of benchmark analysis3

2 Supplementary Figures6

3 Supplementary Tables9

4 References16

## 1 Supplementary Notes

## 1.1 Criteria to define a cell-cell interaction

We will explain the explicit criteria, with examples when it is necessary. If any one of the following conditions applies, we will claim a cell-cell interaction.

1. The experimental evidence includes “co-culture/cultivation/incubation”. For example, “Co-culturing sGFP-expressing HUVEC and aGFP-expressing primary human umbilical vein smooth muscle cells (HUVSMC) resulted in efficient receiver smooth muscle cell labeling” (PMID 33025906) claims an interaction between HUVEC and HUVSMC.

2. The experimental evidence demonstrates that cell type A releases or secretes certain molecules that can be sensed or detected in cell type B. For example, “DC-derived EVs also contribute enzymes for biosynthesis of LTs, key pro-inflammatory mediators important in the pathogenesis of asthma, to smooth muscle cells.” (PMID 32850874) claims DC as the source cell and smooth muscle cells as the target cell.

3. X of cell type A binds to Y of cell type B. For example, “Leptin is a cytokine predominantly secreted by adipose cells and enterocytes in the small intestine and is required for the maintenance of energy homeostasis and body weight (Friedman, 2016). The function of leptin in this process is mediated by binding to the leptin receptor (LEPR) in a subtype of hypothalamic neurons.” (PMID 32822567) claims an interaction from adipose cells to hypothalamic neurons.

4. Physical contact of cell types A and B have a clear functional consequence. For example, “Obese tissues are heavily infiltrated by inflammatory immune cells (e.g., monocytes, macrophages, Th1 cells) which interact with adipocytes to trigger chronic inflammation, ultimately leading to blockage of insulin action on adipocytes and insulin resistance.” (PMID 32486495) claims the interactions from monocytes, macrophages, Th1 cells to adipocytes.

It is difficult to give general simple rules to define the directions, however, it can usually be defined without ambiguity with manual curation. We will explain our point with two examples. Example 1 is an interaction between cardiomyocyte (CM) and endocardial cells (EC). The related sentences are “These results indicated that atrial CM might communicate with ECs in left atrium via NPPA and NPR3 interaction, thereby activating the cGMP-PKG signaling in ECs.” (PMID 2800943). It is clear that CM is the source and EC is the target. Example 2 is an cell-cell interaction mediated by ligand-receptor pairs. The sentences are “Activated Th1 cells release IFNγ, which is a well-known antitumor cytokine that activates macrophage, promotes antigen processing and presentation by APCs, and inhibits angiogenesis” (PMID 31256327). Th1 cell is the source and macrophage is the target, since IFNγ is released by Th1 cell and then it activates macrophage.

## 1.2 An example for querying by cell types

An example for querying by cell types is provided here. If the scientist would like to query about cell-cell interactions involving “epithelial cell”, they can select “epithelial cell” from the root node in the hierarchical trees of cell types and check the option of “Show cell-cell interactions involving cell types of interest” after clicking on the “Reset” button. A figure and table containing 79 interactions involving “epithelial cell” are returned. Users can then choose the “Show cell-cell interactions at the class level” box to figure out which cell types interact with epithelial cell in different contexts from the figure at the class level. If the users are interested in epithelial cell interacting with T cell, they can also obtain epithelial cell interacting with T cell evident in other contexts by clicking on the “Reset” button and selecting the cell types “epithelial cell” and “T cell” from the hierarchical trees of cell types. The query results can tell you that epithelial cell interacting with T cell is experimentally verified in the contexts of inflammatory condition and intestinal epithelium.

## 1.3 Details of benchmark analysis

First, we compared a series of algorithms, namely CellChat [1], CellPhoneDB [2], Connectome [3], iTALK [4], NATMI [5], and SingleCellSignalR [6], and compared them with default LR pairs. The lists of LR pairs and the use of multimeric proteins and agonist/antagonist interactions of different tools are summarized in the Supplementary Table 2.

In default mode, we implemented all algorithms using the resources created by themselves, so that all methods are tested with their full capabilities. Notice that we removed scTensor [7] from the comparison as the algorithm used different criteria for LR pair inclusion, resulting in 45,932 pairs, which is dramatically larger and not comparable to the other algorithms.

We implemented the algorithms in the default parameter setting with the default mode. Details of running each algorithm are illustrated below.

CellChat was run with its default parameter setting where threshold of the p-value for determining significant LR interaction is 0.05. When using CellChat, we did not project gene expression data onto a protein-protein interaction network. CellChat could return a matrix containing the interaction counts and weights between any two cell groups.

CellPhoneDB was run with its default parameter setting where significant (p-value < 0.05) means of the average expression level of ligand and receptor were returned. When using the LR resource created by themselves, the member of the complex with the minimum average expression was considered.

Connectome was run with its default parameter settings where a Wilcoxon Rank test was used to calculate adjusted p-value for ligand and receptor expression and differentially expressed genes (adjusted p-value < 0.05) were kept. Weights of edges in Connectome used two metrics: normalized weight and scaled weight, where scaled weight was used in our comparison analysis just as in the previous study [8].

iTALK was run with its default parameter settings using the ‘mean count’ option which returned the ligand-receptor pairs from top 50% highly expressed genes. The product of mean expression of ligand and receptor in the source and target cell type was used as LR score.

NATMI was run with the default parameter settings. Weights of edges in NATMI used three metrics: mean-expression weight, specificity weight, and total-expression weight, where summed specificity weight was recommended for most analyses as it could capture specific signaling between cell types [5].

SingleCellSignalR was run with the default parameter settings where the log fold-change threshold for differentially expressed genes is 1.5. The LRscores which passed the threshold of 0.9 were used in our comparison analysis. It returned the regularized product LRscore and we pooled paracrine and autocrine interactions.

In summary, each algorithm provides a list of predicted LR interactions with its own format and significance assessment. We summarized the results using the sum of LR scores, recommended in NATMI [5], and the number of significant LR pairs as the strength of cell-cell interactions. Avoided choosing a hard threshold, the prediction results of each method are ranked by its built-in scoring/weighting system. To elaborate, we first rank the predicted cell-cell interactions by the interaction score/weight, and then label the interactions documented in CITEdb as “true”. For the top k interactions, the precision is the number of “true” interactions in the top-k list divided by k. The recall is the number of “true” interactions in the top-k list divided by total number of overlapping interactions between the dataset and CITEdb. By varying k, a precision-recall curve can be obtained for each method. To make it easy for users to reproduce our results and run their own benchmark analysis, we have created an R package (https://github.com/shanny01/benchmark), named benchmark.

It is noteworthy that different approaches to aggregate communication scores for LR pairs can have an impact in the prediction accuracy on the overall state of cell-cell interactions. We also performed the benchmark analysis to combine the tested algorithms with different aggregation approaches. In particular, for directed interactions we used the sum of communication scores (sum method) and the count of the active LR pairs (count method). For undirected interactions, we used Bray-Curtis score [9] and the enrichment score [10]. We are not able to include centrality measures [3] in this comparison, as centrality measures the network property of a node, and it is not straightforward to infer cell-cell interactions from the measure.

We have implemented the benchmark analysis using LIANA [8] for the tested algorithms, including CellChat, CellPhoneDB, Connectome, iTALK, NATMI, and SingleCellSignalR, with the consensus resource created by LIANA. The output LR pairs were unfiltered and then the same threshold as in the default mode were applied to CellChat, CellPhoneDB, Connectome, and NATMI. For iTALK, differentially expressed ligands and receptors with adjusted p-value < 0.05 from Wilcoxon Rank test were kept. For SingleCellSignalR, the LRscores which passed the threshold of 0.2 were used in the comparison analysis. We aggregated the filtered LR pairs of different algorithms from LIANA by the sum of communication scores and the count of active LR pairs. Notice that for iTALK, the mean of logFC of ligand and receptor were calculated as LIANA tutorial described.

When computing Bray-Curtis score and the enrichment score, we first calculated the mean expression for the subset of cells assigned to the corresponding cell types and used an expression threshold over 1 transcripts per million. The consensus resource created by LIANA formed by ligand and/or receptor complexes were transformed to pairwise interactions between individual ligands and receptors. For Bray-Curtis score, of the 21 candidate cell-cell interactions between six cell types (6 self-interactions plus 15 undirected interactions), 15 interactions were documented with experimental evidence at the class level in CITEdb. For the enrichment score, the lists of marker genes for six cell types were retrieved from CellMarker [11]. For the enrichment score, of the 15 candidate undirected cell-cell interactions without self-interactions, 9 interactions were documented with experimental evidence at the class level in CITEdb. Only significantly enriched cell-cell interactions with adjusted p-value < 0.05 were considered in the comparison analysis as [10] suggested.

The precision-recall curve can be obtained as in the default mode. The difference is that regardless of the direction, the interactions documented in CITEdb are labeled as “true” when evaluating Bray-Curtis score and the enrichment score. Plus, we have also evaluated the above-mentioned aggregation approaches in our “benchmark” R package (https://github.com/shanny01/benchmark), allowing for automatic benchmark analysis of directed and undirected cell-cell interactions at overall state level.

Notice that we randomly re-sampled the cell types 100 times for the melanoma dataset. For each random sample, we re-ran the analysis to identify cell-cell interactions, and calculated the precision-call curve and the area under the curve. Subsequently, we calculate the 95% empirical confidence interval of area under the PR curve for all tested methods. To save time, we did not reported 95% empirical confidence intervals for methods in the default mode (Supplementary Figure 2).

**2 Supplementary Figures**


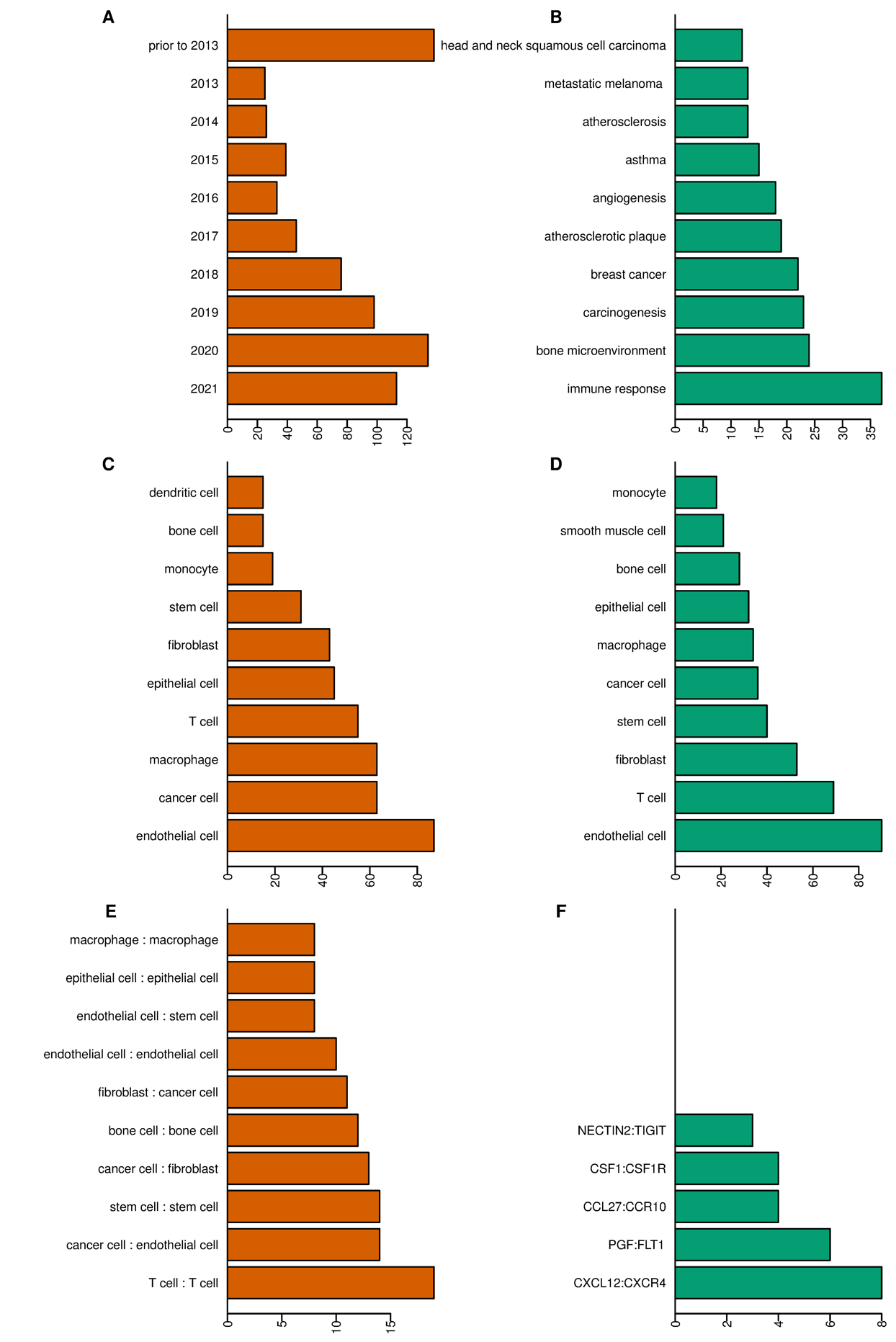


**Supplementary Figure 1.** Statistics of CITEdb. (A) Number of cell-cell interactions reported each year. (B) Number of cell-cell interactions in the top 10 physiological contexts. (C) Number of cell-cell interactions in the top 10 source cell types. (D) Number of cell-cell interactions in the top 10 target cell types. (E) Number of cell-cell interactions in the top 10 pairs of cell types. (F) Number of top ligand-receptor pairs. We first took the subset of cell-cell interactions that are annotated with direction (including bi-directional ones) in CITEdb. Then for each cell type (at class level), we separately counted the number of interactions it being a source and target. Next, the counts were ranked to identify the top cell types.

**
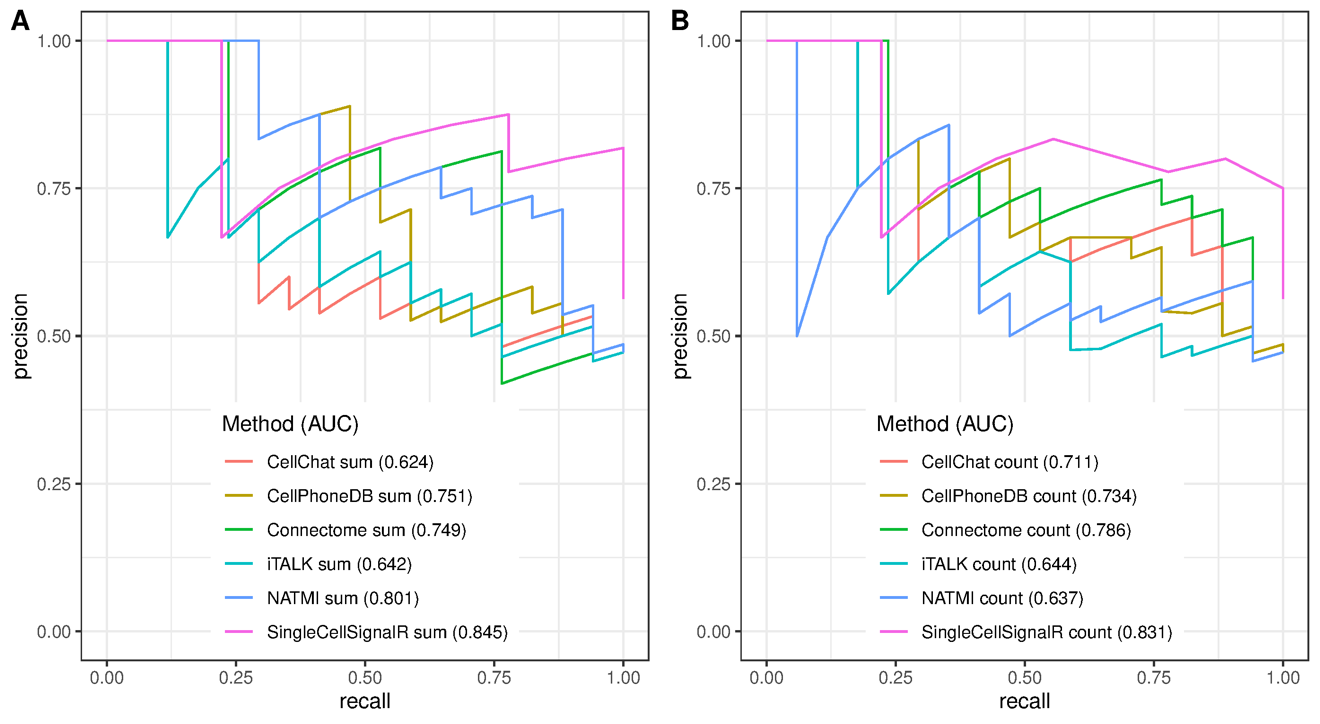
**

**Supplementary Figure 2.** Precision-recall curves in evaluating various algorithms that predict cell-cell interactions from a human metastatic melanoma scRNA-seq dataset. CITEdb interactions are used as a benchmark dataset. Each algorithm is implemented with its default ligand-receptor resources and the results were then summarized into cell-cell interactions by the sum of communication scores (A) and the count of active LR pairs (B).

**3 Supplementary Tables**

**Supplementary Table 1.** Lists of initial references with PMID and those with full text equal to 1 meaning that those were finally considered.

| PMID | Full text | PMID | Full text | PMID | Full text |
| --- | --- | --- | --- | --- | --- |
| 34951683 | 1 | 32257377 | 1 | 28578911 | 1 |
| 34951074 | 1 | 32183816 | 1 | 28326171 | 1 |
| 34936938 | 1 | 32173999 | 1 | 28317948 | 1 |
| 34921143 | 1 | 32153574 | 1 | 28287607 | 1 |
| 34896267 | 1 | 32107314 | 1 | 28285690 | 1 |
| 34864302 | 1 | 32098504 | 1 | 28263321 | 1 |
| 34858875 | 1 | 32059163 | 1 | 28234843 | 0 |
| 34846303 | 1 | 32035230 | 1 | 28230931 | 1 |
| 34831198 | 1 | 32028987 | 1 | 28214945 | 1 |
| 34829933 | 1 | 31915373 | 1 | 28174237 | 1 |
| 34808557 | 1 | 31879153 | 1 | 28077322 | 1 |
| 34768768 | 1 | 31838471 | 1 | 28039134 | 1 |
| 34734037 | 1 | 31825151 | 1 | 27799031 | 0 |
| 34715746 | 0 | 31761445 | 1 | 27744003 | 1 |
| 34685602 | 1 | 31744402 | 1 | 27592803 | 1 |
| 34638604 | 1 | 31733332 | 1 | 27550738 | 1 |
| 34638524 | 1 | 31727590 | 1 | 27521522 | 1 |
| 34623464 | 1 | 31648045 | 1 | 27443883 | 1 |
| 34618691 | 1 | 31646713 | 1 | 27383658 | 0 |
| 34601654 | 1 | 31256327 | 1 | 27111714 | 1 |
| 34597714 | 0 | 31136100 | 1 | 30603452 | 1 |
| 34588551 | 1 | 32464631 | 1 | 28105201 | 1 |
| 34571891 | 0 | 31853340 | 1 | 27807451 | 1 |
| 34566966 | 1 | 31840053 | 1 | 27721433 | 1 |
| 34500367 | 0 | 31835632 | 1 | 27534549 | 1 |
| 34490594 | 0 | 31817101 | 1 | 27492069 | 1 |
| 34456860 | 1 | 31729432 | 1 | 27486976 | 1 |
| 34454076 | 0 | 31671920 | 1 | 27468270 | 1 |
| 34450029 | 1 | 31442885 | 1 | 27324652 | 0 |
| 34445351 | 1 | 31416445 | 1 | 27252357 | 1 |
| 34440172 | 1 | 31397926 | 1 | 27216497 | 1 |
| 34434927 | 1 | 31394194 | 1 | 27215587 | 1 |
| 34422835 | 1 | 31379046 | 1 | 27136321 | 1 |
| 34407376 | 0 | 31348891 | 1 | 27129211 | 1 |
| 34331449 | 1 | 31267326 | 1 | 27101311 | 1 |
| 34327532 | 0 | 31207473 | 1 | 27056720 | 1 |
| 34225662 | 1 | 31192511 | 1 | 26981123 | 1 |
| 34216641 | 1 | 31068444 | 1 | 26967678 | 1 |
| 34130712 | 0 | 31052401 | 1 | 26964503 | 1 |
| 34111027 | 1 | 31034520 | 1 | 26914606 | 1 |
| 34068395 | 1 | 30995076 | 1 | 26912223 | 1 |
| 34030626 | 1 | 30939814 | 1 | 26884279 | 1 |
| 33974001 | 1 | 30934547 | 1 | 26826524 | 0 |
| 33964410 | 1 | 30932215 | 0 | 26764001 | 1 |
| 33963379 | 0 | 30930201 | 1 | 26752061 | 1 |
| 33926561 | 1 | 30895179 | 1 | 26700550 | 1 |
| 33919065 | 1 | 30895170 | 1 | 26472446 | 0 |
| 33885545 | 0 | 30886214 | 1 | 26375517 | 1 |
| 33868170 | 1 | 30857191 | 1 | 24859928 | 1 |
| 33859742 | 1 | 30828948 | 1 | 28936238 | 1 |
| 33808520 | 1 | 30811771 | 1 | 26576206 | 1 |
| 33732657 | 1 | 30774121 | 1 | 26566474 | 1 |
| 33732416 | 1 | 30704133 | 1 | 26544924 | 1 |
| 33729255 | 0 | 30663708 | 1 | 26488876 | 1 |
| 33710972 | 1 | 30659585 | 1 | 26446608 | 1 |
| 33691792 | 1 | 30658653 | 1 | 26398895 | 1 |
| 33687702 | 1 | 30616603 | 1 | 26393296 | 0 |
| 33679763 | 1 | 30612606 | 1 | 26216135 | 1 |
| 33637118 | 1 | 30586277 | 1 | 26204397 | 0 |
| 33604857 | 1 | 30465207 | 0 | 26198319 | 1 |
| 33599250 | 1 | 30324573 | 1 | 26192966 | 1 |
| 33597528 | 1 | 30230353 | 1 | 26159297 | 1 |
| 33572290 | 1 | 32832198 | 1 | 26148937 | 1 |
| 33569058 | 1 | 31719884 | 1 | 26120938 | 1 |
| 33548142 | 1 | 30649646 | 1 | 26079967 | 1 |
| 33542232 | 1 | 30619100 | 1 | 25954876 | 1 |
| 33540650 | 1 | 30569645 | 1 | 25941513 | 1 |
| 33521856 | 1 | 30429548 | 1 | 25900308 | 1 |
| 33513595 | 1 | 30404002 | 1 | 25891384 | 1 |
| 33491924 | 1 | 30402542 | 1 | 25877907 | 1 |
| 33420488 | 1 | 30386101 | 1 | 25869133 | 1 |
| 33416449 | 0 | 30298816 | 1 | 25862842 | 1 |
| 33393143 | 1 | 30280098 | 1 | 25852682 | 1 |
| 33387068 | 0 | 30176009 | 1 | 25840006 | 1 |
| 34691611 | 1 | 30144323 | 1 | 25741775 | 1 |
| 33565978 | 1 | 30008265 | 1 | 25711903 | 1 |
| 33425869 | 1 | 29953723 | 1 | 25633040 | 1 |
| 33332768 | 1 | 29905392 | 1 | 25569160 | 0 |
| 33324545 | 1 | 29902459 | 1 | 25441616 | 1 |
| 33291683 | 1 | 29892006 | 1 | 25203774 | 1 |
| 33244046 | 1 | 29857185 | 1 | 24912785 | 1 |
| 33232386 | 1 | 29855483 | 1 | 25863037 | 1 |
| 33194199 | 1 | 29703082 | 1 | 25531706 | 0 |
| 33158117 | 1 | 29656370 | 1 | 25407601 | 1 |
| 33147626 | 1 | 29615864 | 1 | 25350752 | 1 |
| 33125685 | 1 | 29581472 | 1 | 25315114 | 1 |
| 33092465 | 1 | 29581239 | 1 | 25274756 | 1 |
| 33090076 | 1 | 29566751 | 1 | 25157253 | 1 |
| 33086079 | 1 | 29465752 | 1 | 25130606 | 1 |
| 33080118 | 1 | 29440427 | 1 | 25123278 | 1 |
| 33058349 | 1 | 29425822 | 1 | 25092378 | 1 |
| 33025906 | 1 | 29388137 | 1 | 25059386 | 1 |
| 32981416 | 1 | 29338773 | 1 | 25028490 | 1 |
| 32948210 | 1 | 29222052 | 1 | 24928860 | 1 |
| 32939907 | 1 | 29208635 | 1 | 24924235 | 1 |
| 32899117 | 1 | 29160172 | 1 | 24865867 | 1 |
| 32880912 | 1 | 29095348 | 1 | 24839024 | 0 |
| 32872458 | 1 | 28956068 | 1 | 24831807 | 1 |
| 32850874 | 1 | 28888046 | 1 | 24750670 | 1 |
| 32845745 | 1 | 28611013 | 1 | 24692354 | 1 |
| 32822567 | 1 | 28370277 | 1 | 24584193 | 1 |
| 32800943 | 1 | 28122807 | 1 | 24530222 | 1 |
| 32765582 | 1 | 32161785 | 1 | 24348826 | 1 |
| 32718981 | 1 | 29081734 | 1 | 23980822 | 1 |
| 32678092 | 1 | 29022008 | 1 | 23038605 | 1 |
| 32579974 | 1 | 28901192 | 1 | 25404879 | 1 |
| 32534107 | 1 | 28895404 | 0 | 24564953 | 1 |
| 32486495 | 1 | 28800366 | 1 | 24398998 | 1 |
| 32472127 | 1 | 28774868 | 1 | 24205503 | 1 |
| 32440447 | 1 | 28769044 | 1 | 24130797 | 1 |
| 32363357 | 1 | 28699527 | 0 | 24058665 | 1 |
| 32353936 | 1 | 28684336 | 1 | 24040308 | 1 |
| 32341451 | 1 | 28600910 | 1 | 23963401 | 1 |
| 32258040 | 1 | 28596933 | 1 | 23900167 | 1 |
| 7396358 | 1 | 468376 | 1 | 219769 | 1 |
| 23878230 | 1 | 19629190 | 1 | 16492141 | 1 |
| 23844026 | 1 | 19564624 | 1 | 16381928 | 1 |
| 23840189 | 1 | 19492336 | 1 | 16352326 | 1 |
| 23817070 | 1 | 19462451 | 1 | 16411810 | 0 |
| 23764369 | 1 | 19337375 | 1 | 16166562 | 1 |
| 23707953 | 1 | 19211674 | 1 | 16159901 | 1 |
| 23664885 | 1 | 19208767 | 1 | 15868959 | 1 |
| 23606500 | 1 | 19149601 | 1 | 15725748 | 0 |
| 23562089 | 1 | 19113950 | 1 | 15688009 | 1 |
| 23510904 | 1 | 18837649 | 1 | 15288263 | 1 |
| 23508709 | 1 | 18708174 | 1 | 15265877 | 1 |
| 23472616 | 1 | 19186188 | 1 | 15242756 | 1 |
| 23430333 | 1 | 18757316 | 1 | 15146564 | 1 |
| 23328900 | 1 | 18720445 | 1 | 15094343 | 1 |
| 23278744 | 1 | 18667798 | 1 | 15061569 | 0 |
| 23131042 | 0 | 18652667 | 1 | 15032616 | 1 |
| 23103669 | 1 | 18570454 | 1 | 14997440 | 1 |
| 22244842 | 1 | 18283625 | 0 | 14993837 | 0 |
| 23248599 | 1 | 18262434 | 1 | 14970327 | 1 |
| 23147909 | 1 | 18211261 | 1 | 14691034 | 1 |
| 23109932 | 1 | 18160820 | 1 | 19002943 | 1 |
| 23080410 | 1 | 18082460 | 1 | 14623228 | 1 |
| 23060969 | 1 | 18057119 | 1 | 14612506 | 1 |
| 22957741 | 1 | 18806312 | 1 | 14521518 | 1 |
| 22923488 | 1 | 17956143 | 1 | 12770742 | 1 |
| 22902532 | 1 | 17911156 | 1 | 12594298 | 1 |
| 22886085 | 1 | 17896977 | 0 | 12570929 | 1 |
| 22715383 | 1 | 17887916 | 0 | 12476082 | 1 |
| 22676452 | 1 | 17679141 | 1 | 12566222 | 1 |
| 22515979 | 0 | 17645461 | 1 | 12168060 | 0 |
| 22491366 | 1 | 17634278 | 1 | 12126230 | 1 |
| 22438035 | 1 | 17309823 | 1 | 12000221 | 0 |
| 22433991 | 1 | 16968164 | 0 | 11934257 | 1 |
| 22410558 | 1 | 16931449 | 0 | 11711124 | 1 |
| 22363483 | 1 | 16845172 | 1 | 11787861 | 0 |
| 22360529 | 1 | 16814859 | 1 | 11768200 | 1 |
| 22285593 | 1 | 16799204 | 0 | 11683185 | 1 |
| 22222464 | 1 | 19867118 | 1 | 11641219 | 1 |
| 22210865 | 1 | 8581143 | 1 | 11496230 | 1 |
| 21745193 | 1 | 8557754 | 1 | 11465535 | 0 |
| 22393462 | 1 | 7884860 | 1 | 11380426 | 1 |
| 22216308 | 1 | 7875205 | 1 | 11336798 | 1 |
| 22112782 | 1 | 7697810 | 1 | 11333207 | 0 |
| 22041191 | 1 | 7627716 | 0 | 11245428 | 1 |
| 22012735 | 1 | 8313516 | 1 | 11238529 | 1 |
| 21983923 | 1 | 8291606 | 1 | 11145206 | 1 |
| 21962080 | 1 | 8029502 | 1 | 11121235 | 1 |
| 21915303 | 1 | 8027041 | 1 | 11229597 | 0 |
| 21847077 | 1 | 7949103 | 0 | 11144406 | 1 |
| 21731625 | 1 | 7533044 | 0 | 10952915 | 1 |
| 21720525 | 1 | 7516973 | 1 | 10773588 | 1 |
| 21708226 | 1 | 20692978 | 0 | 10699360 | 1 |
| 21674053 | 1 | 8406571 | 1 | 10615428 | 1 |
| 21656608 | 1 | 8388962 | 0 | 10615413 | 1 |
| 21621608 | 1 | 8362976 | 1 | 10548431 | 1 |
| 21592329 | 1 | 8320264 | 1 | 10460003 | 1 |
| 21585313 | 1 | 8097047 | 1 | 10403569 | 1 |
| 21515906 | 1 | 1454206 | 0 | 10065948 | 0 |
| 21405953 | 0 | 2064213 | 1 | 9876332 | 1 |
| 21342503 | 1 | 2022665 | 1 | 9694044 | 0 |
| 21183475 | 1 | 1902631 | 1 | 9628901 | 1 |
| 21044087 | 1 | 2236758 | 0 | 9617570 | 1 |
| 20876208 | 1 | 2224131 | 0 | 9535221 | 1 |
| 21057637 | 1 | 2160322 | 1 | 9496677 | 0 |
| 20728208 | 1 | 2119362 | 1 | 9472694 | 1 |
| 20603896 | 1 | 2112060 | 1 | 21781865 | 1 |
| 20419629 | 1 | 2677029 | 1 | 9434160 | 1 |
| 20167338 | 1 | 2475187 | 0 | 9409458 | 1 |
| 20147621 | 1 | 2895712 | 1 | 9358032 | 0 |
| 20144913 | 1 | 3782029 | 1 | 9271306 | 1 |
| 20126618 | 1 | 4041617 | 0 | 9250160 | 1 |
| 19998330 | 1 | 6468760 | 0 | 9153270 | 1 |
| 19907980 | 0 | 6872157 | 1 | 8909254 | 1 |
| 19796498 | 1 | 6826646 | 1 | 8831287 | 1 |
| 19274711 | 1 | 7263074 | 1 | 8781343 | 1 |
| 19774227 | 1 | 19637986 | 1 | 8695757 | 0 |
| 8675597 | 1 |  |  |  |  |

**Supplementary Table 2.** Lists of LR pairs in different tools.

| Tools | Language | LR size | Multimeric proteins | Agonist/antagonist interactions |
| --- | --- | --- | --- | --- |
| CellChat | R | 1,939 | Yes | Yes |
| CellPhoneDB | python | 1,396 | Yes | No |
| Connectome | R | 2,557 | No | No |
| iTALK | R | 2,649 | No | No |
| NATMI | python | 2,293 | No | No |
| SingleCellSignalR | R | 3,251 | No | No |
| scTensor | R | 45,932 | No | No |

## References

[1] Jin, S., et al. Inference and analysis of cell-cell communication using CellChat. Nat Commun 2021;12(1):1088.

[2] Efremova, M., et al. CellPhoneDB: inferring cell-cell communication from combined expression of multi-subunit ligand-receptor complexes. Nat Protoc 2020;15(4):1484-1506.

[3] Raredon, M.S.B., et al. Computation and visualization of cell-cell signaling topologies in single-cell systems data using Connectome. Sci Rep 2022;12(1):4187.

[4] Wang, Y., et al. iTALK: an R Package to Characterize and Illustrate Intercellular Communication. bioRxiv 2019.

[5] Hou, R., et al. Predicting cell-to-cell communication networks using NATMI. Nat Commun 2020;11(1):5011.

[6] Cabello-Aguilar, S., et al. SingleCellSignalR: inference of intercellular networks from single-cell transcriptomics. Nucleic Acids Res 2020;48(10):e55.

[7] Tsuyuzaki, K., Ishii, M. and Nikaido, I. Uncovering hypergraphs of cell-cell interaction from single cell RNA-sequencing data. bioRxiv 2019:566182.

[8] Dimitrov, D.*, et al.* Comparison of Resources and Methods to infer Cell-Cell Communication from Single-cell RNA Data. Nat Commun 2022;13(1):3224.

[9] Armingol, E., et al. Inferring a spatial code of cell-cell interactions across a whole animal body. bioRxiv 2022:2020.2011.2022.392217.

[10] Krausgruber, T., et al. Structural cells are key regulators of organ-specific immune responses. Nature 2020;583(7815):296-302.

[11] Zhang, X., et al. CellMarker: a manually curated resource of cell markers in human and mouse. Nucleic Acids Res 2019;47(D1):D721-d728.
